# New closed tube loop mediated isothermal amplification assay for prevention of product cross-contamination

**DOI:** 10.1016/j.mex.2014.08.009

**Published:** 2014-08-27

**Authors:** K. Karthik, Rajesh Rathore, Prasad Thomas, T.R. Arun, K.N. Viswas, Kuldeep Dhama, R.K. Agarwal

**Affiliations:** a Division of Bacteriology and Mycology, Indian Veterinary Research Institute, Bareilly 243122, India; b Division of Pathology, Indian Veterinary Research Institute, Bareilly 243122, India

**Keywords:** LAMP, *Brucella*, Contamination, Agar dye capsule, LED, SYBR green

## Abstract

Loop mediated isothermal amplification (LAMP) assay, a promising diagnostic test, has been developed for detection of different pathogens of human as well as animals. Various positive points support its use as a field level test but the major problem is product cross contamination leading to false positive results. Different methods were adopted by various researchers to control this false positive amplification due to cross contamination but all have their own advantages and disadvantages. A new closed tube LAMP assay based on agar dye capsule was developed in the present study and this technique has some advantages over the other closed tube technique.

• Agar at the concentration of 1.5% was used to sandwich SYBR green dye I with the aid of intradermal syringe. This agar dye capsule was placed over the LAMP reaction mixture before it was amplified.
• To eliminate the hazardous nature of Ultra Violet (UV) light during result visualization of LAMP products, the present study demonstrates the use of Light Emitting Diode (LED) lights for result visualization.
• LAMP was carried out for *Brucella* species detection using this modified techniques yielding good results without any cross contamination and LED showed similar fluorescence compared to UV.

## Graphical abstract

**Figure:**
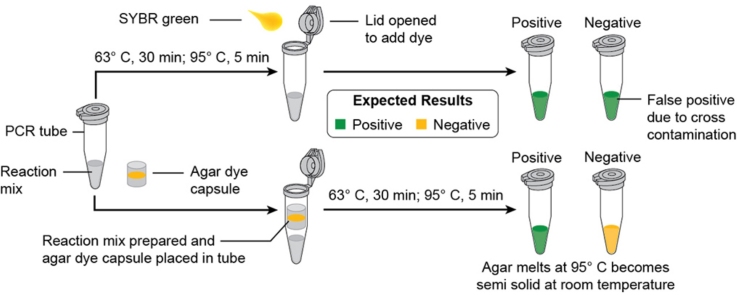

## Method details

The present work was designed to develop a closed tube LAMP assay using agar dye capsule using SYBR green dye I, having good sensitivity and also the use of Light Emitting Diode (LED) bulbs to fluoresce dye instead of UV light. LAMP assay for detection of *Brucella* spp. was designed using this modified closed tube method combined with LED technique for result visualization.

### Optimization of LAMP assay for *Brucella* spp.

Three *Brucella* species (*B. abortus* S99, *B. abortus* S19, *B. melitensis*, *B. suis*) and 11 non-*Brucella* species were used in the present study for optimization and also to assess specificity of LAMP assay (Table 1).

LAMP primers were designed using primer explorer online software version 4 for the detection of *Brucella* spp. targeting *Omp*25 gene. F3 and FIP primers were modified from the earlier report of Lin et al. [1] so as to accommodate loop primers. Loop primers were designed using same software in order to see the effect of loop primers on reaction time. Sequences of the primers used are presented in the Table 2. LAMP reaction conditions were adopted as per Lin et al. [1] and optimization for time, temperature and for different chemicals were carried out. PCR targeting *bcsp*31 gene of *Brucella* was used alongside to compare the results with LAMP [2].

The 25 μl final volume of LAMP mixture contained 5 pM of each outer primer (F3 and B3), 40 pM of each inner primer (FIP and BIP) and 20 pM of each loop primer (LF and LB). Thermopol buffer 2.5 μl [1× buffer comprised of 25 mM Tris–HCl pH 8.8, 12.5 mM KCl, 12 mM (NH_4_)_2_SO_4_ and 0.125% Tween 20 (New England Biolabs, USA)], 8 mM MgSO_4_, 1.2 mM dNTPs, 1 M Betaine were the other components in the reaction along with nuclease free water (NFW) to make the volume to 22 μl. To the mixture 2 μl of standard *Brucella abortus* S99 (Reference diagnostic strain) DNA template was added and the mixture was denatured at 95 °C for 5 min. Then the mixture was chilled on ice, finally *Bst* DNA polymerase (8 Units) [Sigma, USA] was added and the reaction was incubated at 63 °C for 30 min. After 30 min the reaction was kept at 95 °C for 5 min to inactivate the enzyme. Results were interpreted by electrophoresis or by adding SYBR green dye I after amplification.

Sensitivity of LAMP assay was carried out with standard *B. abortus* S99 DNA which was serially diluted having an initial concentration of 700 ng/μl. The results were compared with PCR. Similarly specificity of LAMP was also calculated with the bacterial DNA mentioned in Table 1. Cattle whole blood samples (*n* = 200) from field were collected for testing the LAMP assay and the results were compared with PCR. DNA from the samples was extracted as per Sambrook and Russell [3] protocol. The LAMP assay optimized was specific only to *Brucella* spp. and not to any other pathogen targeted in the study. Use of loop primers has reduced the reaction time to 30 min. Sensitivity of LAMP assay with loop primers was 10-fold higher compared to PCR. LAMP assay detected 17 field samples to be positive for *Brucella* spp. while PCR detected only 15 samples to be positive for *Brucella* ssp. All the procedures were carried out under laminar air flow. Screening of 200 cattle whole blood samples was carried out batch wise where each batch had 8 whole blood DNA samples, 1 negative control (no template) and 1 positive control (Known *B. abortus* S99 DNA). After screening 50 samples, there was an occasion when all 10 samples gave positive result including negative control. Further screening of samples had the similar problem of positive results in all samples. The problem was later found to be LAMP product cross contamination which might have occurred while opening the lid after amplification to add SYBR green dye. LAMP product was stable and once the product contaminates the working area or the chemical used further screening will always yield positive results. To avoid this, preventing the opening of lid after amplification is the only way. In order to sort out this problem a new agar dye capsule based closed tube LAMP assay was developed.

### Preparation agar dye capsule

**Fig. 1:**
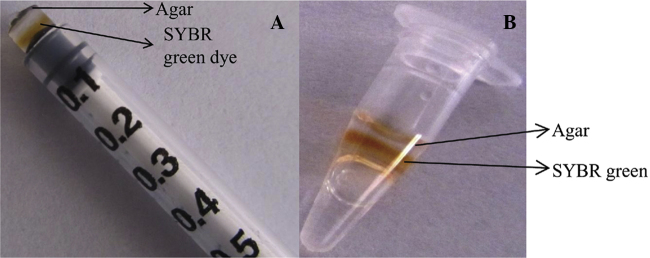
Agar dye capsule preparation. (A) Sandwiching SYBR green dye in agar with the help of intradermal syringe. (B) Positioning of agar dye capsule in the PCR tubes.

**Fig. 2:**
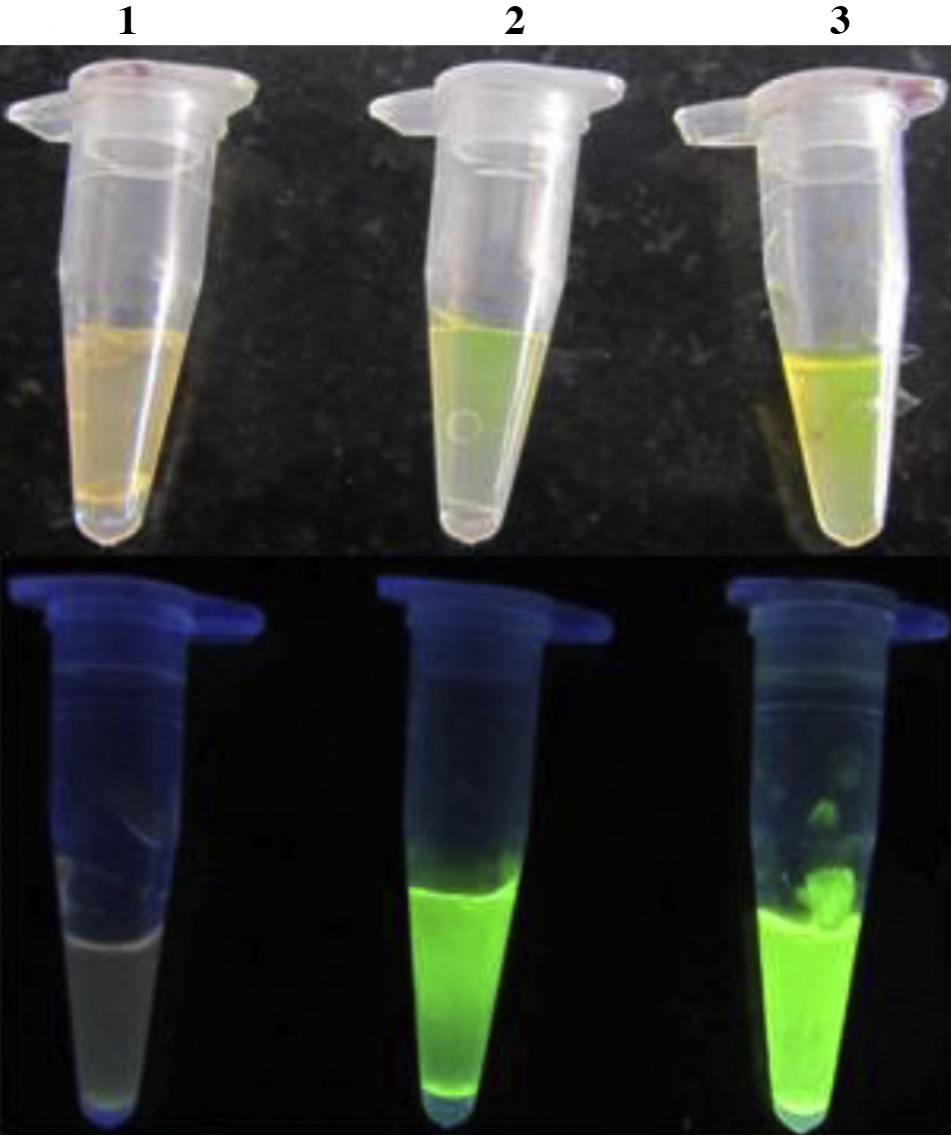
Optimization of SYBR green in the agar dye capsule. Top figure – day light, Bottom figure – under UV light. Tube 1 – 1 μl, Tube 2 – 2 μl, Tube 3 – 3 μl of SYBR green.

1. Agar (Hi Media, India) 1.5% was prepared using autoclaved distilled water and was allowed to cool. The agar used was pure and the water used was also sterile.
2. The barrel tip of 1 ml intradermal syringe was cut to expose plunger tip.
3. Molten agar of 20 μl quantity was loaded into the intradermal syringe through the cut site so that it overlays the plunger tip and was allowed to solidify for 1–2 min.
4. Over the solid agar 2 μl of SYBR green was loaded.
5. Over the SYBR green another layer of 20 μl of molten agar was added and allowed to solidify (Fig. 1).
6. LAMP reaction mix was prepared in PCR tubes as mentioned earlier.
7. The capsule was plunged into the PCR tubes which were already with LAMP reaction mixture. The capsule was kept in a position such that it should not touch the cap of PCR tube and also it should not touch the reaction mixture (Fig. 1).
8. Usual LAMP reaction was carried out at the optimized temperature and the final inactivation was done at 95 °C for 5 min because agar can melt easily at this temperature.
9. The quantity of SYBR green to be kept in the capsule was optimized with various quantity of SYBR green dye and finally it was found that both 2 and 3 μl were found to yield good results (Fig. 2). We used 2 μl SYBR green throughout the study.

### Advantages of agar dye capsule technique

The advantages of agar over wax dye method reported by earlier workers [4–6] are (1) agar is easily available, (2) agar can withstand high temperature so it can hold SYBR green in the capsule form during the course of the reaction and releasing it when the temperature is raised, and (3) once the agar melts and the SYBR green mixes with product, agar becomes semi-solid as it is brought to the room temperature and hence there was no chance of spillage or vaporization of final LAMP products. Similarly, the use of tin foil method by Hong et al. [5] needs some modification in the tubes used for LAMP assay and finally the tubes have to be centrifuged for allowing the SYBR green to react with product. These additional steps were not required in agar dye capsule technique. Though metal indicators like HNB and calcein can be used at the start of the reaction making the system closed but several workers found that SYBR green has good sensitivity than other dyes and metal indicators [7,8]. SYBR green cannot be used at the start of the reaction into the mix as it inhibits the reaction [9]. So to get good sensitivity combined with closed tube method the present study was carried out. Using this agar dye capsule technique, 50 field samples (17 positive and 33 negative samples) which were previously screened by LAMP and PCR were used to know its efficacy. There was no change in the results in LAMP with agar dye capsule and without agar dye capsule. One disadvantage with this agar dye capsule method is that the capsule cannot be stored at −20 °C for longer period because agar will shrink at that temperature; hence it has to be prepared before the reaction mix preparation and can be kept at room temperature till the reaction mix is prepared or at 4 °C for a day.

### Light Emitting Diode (LED) light based LAMP assay result visualization

**Fig. 3:**
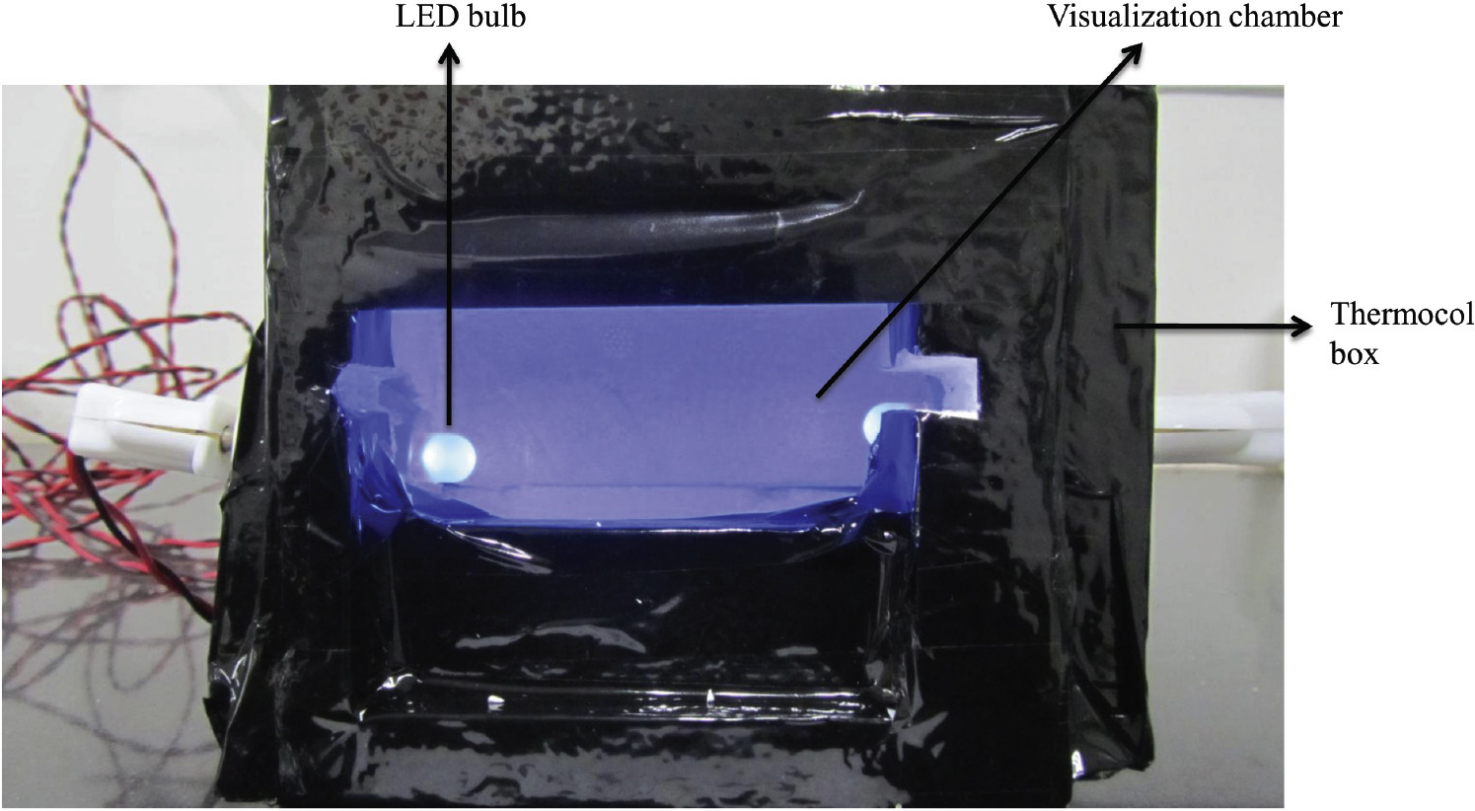
LED light box used for result visualization.

**Fig. 4:**
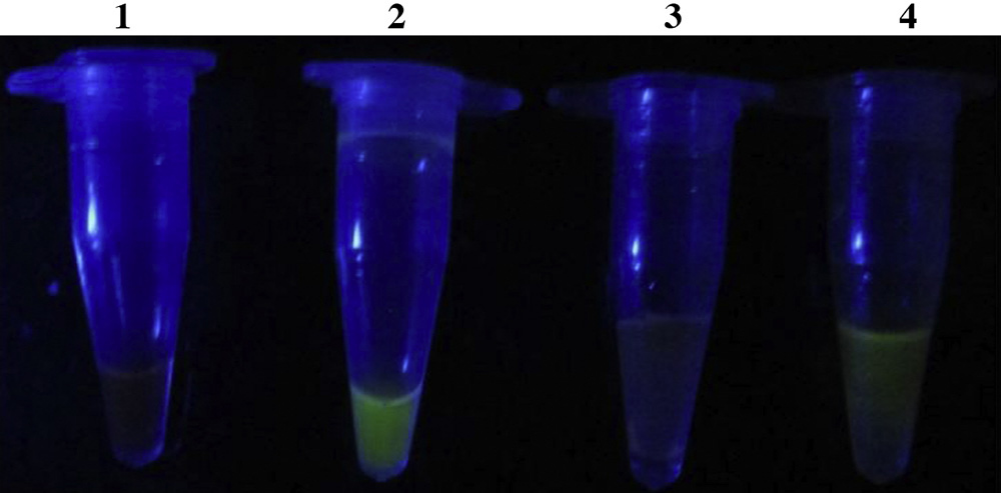
Result visualization under LED light. Tube 1 – Negative; Tube 2 – Positive (without agar dye capsule). Tube 3 – Negative; Tube 4 – Positive (with agar dye capsule).

1. LED light was also used to evaluate its potential to replace UV light for fluorescence of SYBR green allowing result visualization of LAMP products.
2. A thermocol cubical box of 15 cm × 15 cm × 15 cm size was made and two LED bulbs (purchased from local markets) were inserted into the box from the sides.
3. Six slots were made in the top of the box so that 6 PCR tubes can be accommodated and the LED bulbs were positioned so that it will be perpendicular to the PCR tubes.
4. To interpret the results a visualization chamber is provided in front to view the fluorescence and the LED bulbs were connected to electrical circuits through wires (Fig. 3). To make it portable a modification was also done to the box, a 4-volt rechargeable battery was connected in the circuit which can provide the required current to the LED bulbs. The fluorescence under LED light was similar to UV fluorescence (Fig. 4). UV is hazardous to health [10] and hence the alternative LED system can replace it and also the cost of LED lights is cheaper compared to UV light. One more advantage of this LED box is that fluorescence can be visualized even without darkness as the box provides the required darkness for better visibility.

In conclusion LAMP assay is fast, specific, sensitive and reliable test for the detection of *Brucella* spp. at field level. Combination of agar dye capsule and LED based LAMP can help to avoid contamination and make result visualization easy even at field level. This agar dye capsule technique can be carried out for LAMP assays for different pathogen so as to eliminate contamination problem in LAMP assay making the assay applicable at field level.

## Additional information

### Background

Loop mediated isothermal amplification assay (LAMP) has made diagnosis simple at field level because of its swiftness, sensitivity, specificity and adaptability. LAMP has been developed for many pathogens with good success as a diagnostic tool compared with other molecular techniques [11]. LAMP assay has numerous advantages like working at constant temperature, no need of sophisticated instruments, no need of post amplification modification, good sensitivity and specificity hence it could easily be used as a diagnostic assay at field level. LAMP still has some drawbacks like product cross contamination which is a major issue because all samples including negative control show positive and it has been reported by many workers [12–15]. The reason for this cross contamination has not been elucidated properly but chance of contamination is more when the lids of the reaction tubes are opened at the end of the reaction to add dye for result visualization [14]. Few studies reported closed tube result visualization for LAMP to minimize contamination [4–6]. Earlier workers used wax dye capsule method, tin foil method and some used calcein, hydroxy napthol blue metal indicators which can be used at the start of the reaction so that opening of lid after amplification is prevented [16]. SYBR green dye I was found to have good sensitivity and good clarity in result visualization over metal indicators [7,8]. Hence SYBR green was superior to other metal indicators and used for LAMP product visualization. SYBR green fluoresce in presence of Ultra Violet (UV) light, the property which is used for LAMP result visualization. SYBR green can also fluoresce in blue light having a wave length of 475–495 nm [4]. The property of which was exploited in the present study.

## Figures and Tables

**Table 1:** Bacterial isolates used.

S. no.	Bacterial isolates	Source
1	*Brucella abortus* S19 (vaccine strain)	Division of Biological Standardization
2	*Brucella abortus* S99 (diagnostic reference strain)	Division of Biological Standardization
3	*Brucella melitensis* 16 M	Division of Veterinary Public Health
4	*Brucella melitensis* field isolate	General Bacteriology Lab
5	*Brucella suis*	Division of Veterinary Public Health
6	*Escherichia coli*	Division of Veterinary Public Health
7	*Salmonella* Typhimurium	Division of Veterinary Public Health
8	*Yersinia enterocolitica*	Division of Veterinary Public Health
9	*Pasteurella multocida* B:2	Division of Bacteriology and Mycology
10	*Clostridium chauvoei*	Division of Bacteriology and Mycology
11	*Clostridium perfringens*	Division of Bacteriology and Mycology
12	*Staphylococcus aureus* field isolate	General Bacteriology Lab
13	*Campylobacter jejuni* field isolate 1	Division of Veterinary Public Health
14	*Campylobacter jejuni* field isolate 2	Division of Veterinary Public Health
15	*Listeria monocytogenes*	Division of Veterinary Public Health
16	*Shigella flexneri*	Division of Veterinary Public Health

**Table 2:** LAMP primers used.

S. no.	Name	Sequence (5′–3′)	Reference
1	F3	ATCCAGGAACAGCCTCCG	Self-designed
2	B3	GCATCACCTTCAACACCGTA	Lin et al. [1]
3	FIP	TTGTTCCAGCCATAGCCAAGGTGGTTGAAGTAGCTCCCCA	Self-designed
4	BIP	CAGCACCGTTGGCAGCATCATCTGGTCCTGCTGGAAGTT	Lin et al. [1]
5	LF	ATAGCCACCAGCCCAGCTATA	Self-designed
6	LR	AAGGCTGGCGCCTTTGCTG	Self-designed
